# The relationship between perceived stress and bedtime procrastination among Chinese college students: a moderated mediation model

**DOI:** 10.3389/fpsyt.2025.1547389

**Published:** 2025-04-09

**Authors:** Qiuyue He, Huiyi Wu, Xiaolu Meng, Chunlu Li

**Affiliations:** ^1^ Department of Psychology, School of Medical Humanitarians, Guizhou Medical University, Guiyang, China; ^2^ Guizhou Health Development Research Center, Guiyang, China; ^3^ Department of Histology and Embryology, School of Basic Medical Sciences, Guizhou Medical University, Guiyang, China; ^4^ Key Laboratory for Research on Autoimmune Diseases of Higher Education Schools in Guizhou Province, Guiyang, China

**Keywords:** perceived stress, bedtime procrastination, life history strategy, distress tolerance, Chinese college students

## Abstract

**Introduction:**

the role of stress in inducing bedtime procrastination is a new research topic that has emerged in recent years. This study aimed to explore the psychological mechanism by which perceived stress affects bedtime procrastination in Chinese college students in a life history framework.

**Methods:**

first, we investigated whether life history strategy mediate their relationships. Then, we examined whether distress tolerance moderated the direct effects of perceived stress on bedtime procrastination and/or the indirect effects of them mediated by life history strategy. The data of 1021 college students were analyzed.

**Results:**

the results showed that: (1) perceived stress had a significant predictive effect on bedtime procrastination; (2) life history strategy played a mediating role in the relationship between perceived stress and bedtime procrastination; (3) distress tolerance moderated the indirect effect between them mediated by life history strategy, but not the direct effect between them.

**Discussion:**

the present study suggested that life history-based interventions might be an effective intervention for stress-induced bedtime procrastination. Specifically, it might be helpful for alleviation of bedtime procrastination to develop slow life history strategy, when faced with similar stressful situations in the future. Furthermore, distress tolerance may be an important alternative target.

## 1 Introduction

Bedtime procrastination is defined as frequently failing to go to bed at the intended time, while no external circumstances prevent a person from doing so (1). The prevalence of bedtime procrastination among college students is higher than that among non-students (2). A survey conducted by China Youth Daily in 2019 among more than 1,000 college students showed that more than 30% of them often stayed up late and slept less than 7 hours a night (3). Bedtime procrastination not only causes sleep insufficiency (4), affects an individual’s learning and working status the next day (5, 6), but also reduces individual well-being and increases risk of physical and mental illness, such as anxiety and depression (7, 8). Given the high prevalence and serious consequences, identifying the influencing factors and psychological mechanisms of bedtime procrastination among Chinese college students is crucial for effective intervention.

Except for a few studies focusing on time perspective, such as chronotype (9, 10) and future time perspective (9, 11), a large number of previous studies have mainly understood bedtime procrastination among college students from the perspective of self-regulation. Bedtime procrastination is often conceptualized as a consequence of poor self-regulation (5) and its associated disorders, such as mobile phone addiction (12–19).

However, these studies on college students’ bedtime procrastination did not pay enough attention to the special period of their lives. College students are most in emerging adulthood, which roughly ages 18–25 (20). This period is considered to be a critical and most unstable period of their life span, with many life transitions in terms of living arrangements, relationships, education and employment (21). On the other hand, their ability to deal with problems in life is still immature (22). As a result, college students are believed to have a high level of perceived stress. This is especially true for Chinese college students, because they often the only children in their families and thus are very sensitive to stress. In fact, studies have shown that Chinese college students have high levels of stress (23, 24). A recent qualitative study of a sample of adolescent students found that stress is a highlighted factor associated with bedtime procrastination (25). However, the impact of perceived stress on bedtime procrastination among Chinese college students and its psychological mechanisms are still not fully understood.

A recent study of a Chinese college student sample explored the relationship between academic stress and bedtime procrastination (26). They found that academic stress positively predicted bedtime procrastination, and mobile phone addiction was an important mediator between the two variables. According to the stressor-detachment model, they speculated that mobile phone addiction is a mean of detachment for recovery under academic workload. From this perspective, mobile phone addiction behavior before bedtime is an active strategy, while bedtime procrastination is an active procrastination behavior. In line with this, a qualitative study of new career starters under the pressure of the university-to-work transition found that leisure activities before bedtime is an active behavior for me-time and self-negotiation (27). This is clearly inconsistent with the view that bedtime procrastination is a consequence of self-regulation failure. From the perspective of self-regulation failure, bedtime procrastinator cannot resist the temptation and are more likely to involve in entertaining activities before bedtime at night, then bedtime procrastination seems to be a passive behavior rather than an active procrastination behavior. Therefore, the self-regulation failure model cannot well explain the bedtime procrastination induced by perceived stress.

Why bedtime procrastinators actively choose non-adaptive behaviors over adaptive behaviors is a puzzling and critical question that urgently needs to be explained. One scholar has tried to use the perspective of resource conflict to explain the tension between leisure and health in bedtime procrastination, that is, individuals want to satisfy both pleasure or leisure needs and health needs, but one of the needs is at the expense of the other, and they are competing for the same time resource (28). While bedtime procrastinators appear to focus on the present rather than the future in their decision-making about handling this conflict. For example, the future time perspective negatively predicts bedtime procrastination among college students sample (9, 11). Performance goal orientation, a future time orientation disposition, also negatively predicted bedtime procrastination (3). The viewpoints of resource competition, future time perspective and active strategy are highly consistent with the life history strategy (LHS) perspective in evolutionary psychology, suggesting that the life history strategy perspective may help integrate these perspectives and better understand this phenomenon, especially for college students who are in emerging adulthood, a period of heightened instability.

### 1.1 The mediating role of life history strategies in the relationship between perceived stress and bedtime procrastination

LHS focuses on how individuals allocate their limited resources to adapt to stressful and challenging environments based on an assessment of the environmental constraints (29). When the environment is predictable, planning and working towards higher future returns is cost-effective. Therefore, humans and animals’ cognition and behavior tend to be more future- than present-oriented. That is, they prefer actions that may yield high future rewards but have no or little immediate benefits. This is called the slow LHS. Within the same framework, people with slow LHS are expected to be future-oriented and not procrastinate (30). Conversely, when the future is uncertain and difficult to predict, the likelihood of an investment paying off in the future is slim. Present orientation has greater practical significance for individual survival than future orientation. Therefore, organisms will pay more attention to the present and discount the future. This is called the fast LHS. Since investing in the present is most profitable compared to the future, people who adopt a fast LHS are expected to procrastinate (30).

Environmental stress is a key antecedent factor in inducing a fast life history strategy (31). In human life history strategies, subjective perceived stress can more accurately reflect an individual’s stress state than objective environmental stress and can more effectively induce a fast life history strategy. Perceived stress refers to the imbalance between perceived environmental demands and their ability to cope with those demands (32). When an individual believes that his or her abilities are adequate to cope with environmental demands, perceived stress is low. Conversely, when an individual believes that his or her abilities are insufficient to cope with environmental demands, perceived stress is high. Chinese college students, who are often the only children in their families, are very sensitive to stress. They face multiple pressures, such as changes of environmental and lifestyle, academic workload, and interpersonal relationships. In fact, studies have shown that Chinese college students have high levels of stress (23, 24). Existing evidences showed that perceived stress is closely related to bedtime procrastination among Chinese college students. For example, perceived stress was significantly positively correlated with bedtime procrastination (33). Self-efficacy, the subjective feeling of ability to handle stress, an important antecedent of perceived stress, was negatively associated with bedtime procrastination (34).

After experiencing stress during the day, college students are faced with a dilemma between leisure and health (28), which involves how to optimally allocate limited time resources to maximize adaptation to the stressful environment. According to the concept of perceived stress, higher perceived stress often means a greater lack of ability to cope with environmental demands, which not only means more resource depletion, but also indicates a loss of control over future outcomes and increased uncertainty about the future. In this context, for those with higher perceived stress, their future is uncertain and difficult to predict, and the possibility of getting a return on investment in the future is smaller. Therefore, it is more important for them to recover from the individual fatigue induced by excessive stress. Detachment from stressful events might be the best way to recover. The increase in the demand for me-time and self-negotiation may reflect this need for detachment. That might be a need for work-life balance, specifically the balance between “what I want to do” and “what I am expected or should do”. This balance is critical to the well-being of college students (35, 36) and a predictor of college student anxiety and depression (37). That is, under stressful situations, a fast life history strategy that focuses on the present and discounts the future is more adaptive to the environment. Therefore, it is reasonable to hypothesize that LHS mediates the effects of perceived stress on bedtime procrastination (H1).

### 1.2 The moderation of distress tolerance

If the aforementioned hypothesis 1 is true, how to alleviate perceived stress-induced bedtime procrastination based on this model is a topic worthy of further exploration. Perceived stress is often accompanied by negative physical and emotional states. The ability to effectively withstand these aversive internal experiences, termed distress tolerance (DT), has long been of interest in psychotherapy (38–40). Distress intolerance is an important motivator of maladaptive avoidance-based coping strategies (41). Low DT has been associated with maladaptive avoidance behaviors, such as procrastination behavior (42), substance use (43, 44), stock addiction (45), depression and anxiety (46, 47).

On the one hand, individuals with low distress tolerance have been reported to have greater responses to distress and are more likely to exceed their tolerance threshold and experience stress overload because of their low distress tolerance threshold (48). On the other hand, distress intolerance may motivate these self-defeating behaviors, serving as powerful proximal reinforcers to quickly relieve distressing states interpreted as dangerous or unmanageable (41). These active choices by individuals may arise from their belief that they cannot or do not want to endure present pain in the future (49). In this context, individuals with low distress tolerance are expected to have a stronger need to relieve negative emotions and be more likely to initiate the fast LHS and/or indulge in relaxing activities before going to bed, which can lead to delayed bedtime. Therefore, distress tolerance appears to moderate the relationship between perceived stress and LHS (H2) and the relationship between perceived stress and bedtime procrastination (H3).

**Figure 1:**
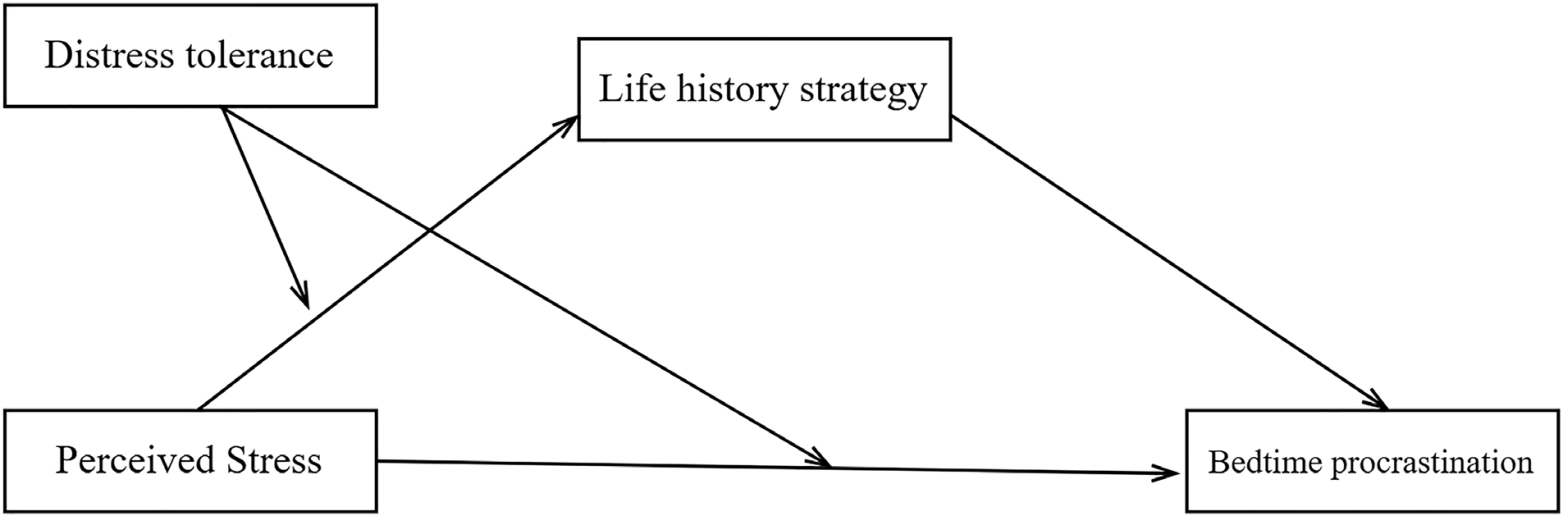
The moderated mediation model hypothesized in the present study. Notes of Figure  In this model, we hypothesized that LHS may mediate the relationship between perceived stress and bedtime procrastination (H1); and that distress tolerance may moderate both the relationship of perceived stress and life history strategy (H2) and that of perceived stress and bedtime procrastination (H3).

In summary, this study aimed to explore the psychological mechanism by which perceived stress affects bedtime procrastination in Chinese college students. First, we investigated whether life history strategy mediates their relationships. Then, we examined whether distress tolerance moderated the direct effects of perceived stress on bedtime procrastination and/or the indirect effects of them mediated by life history strategy. **Figure 1** shows the hypothesized overall model.

## 2 Methods

### 2.1 Participants and procedure

We recruited Chinese college students from three universities in Guizhou, Shandong, and Fujian provinces, which are located in the southwest, north and east of China respectively. Prior to participation, all students signed informed consent forms and were informed that their responses would be anonymous with the option of withdrawal at any time.1021 students were included in the data analysis. Among them were 335 males and 686 females; 612 were ethnic minorities and 409 were Han Chinese; 247 were from towns and 774 were from villages; 423 had left-behind experience; 598 had no left-behind experience. The participants ranged in age from 18 to 22 years old, and the average age was 18.97 ± 0.96 years. This research was approved by the ethics committee of Guizhou Medical University.

### 2.2 Measures

#### 2.2.1 Bedtime procrastination

The Bedtime Procrastination Scale (50) was used to measure bedtime procrastination. The Chinese version of the Bedtime Procrastination Behavior Scale has good reliability and validity in college students. It consists of nine items, such as “I sleep later than I expected.” and the responses of participators were rated on a 5-point Likert scale, ranging from 1 (never) to 5 (always). Items 2, 3, 7, and 9 were scored in reverse. Higher scores indicated more bedtime procrastination behaviors (51). Cronbach’s alpha of this scale was 0.80 in the present study.

#### 2.2.2 Perceived stress

The Perceived Scale (52) was used to measure stress. The Chinese version of the Perceived Stress Scale has been demonstrated to have good reliability and validity. It contains 14 items, such as “I feel out of control of the important things in my life” and the responses of participators were rated on a 5-point Likert scale ranging from 1 (strongly disagree) to 5 (strongly agree). Higher scores indicated higher perceived stress (53). Cronbach’s alpha was 0.75 in the present study.

#### 2.2.3 Life history strategy

LH strategy was measured using the Mini-K Life History Strategy Scale (54). The Chinese version of the Mini-K Life History Strategy Scale demonstrated good reliability and validity among Chinese College Students. It consists of 19 items, such as “I try to understand how I got into a situation to figure out how to handle it.” The responses of participators were rated on a 7-point Likert scale ranging from 1 (strongly disagree) to 7 (strongly agree). Higher scores indicated a slower life history strategy (55). Cronbach’s alpha was 0.80 in the present study.

#### 2.2.4 Distress tolerance

The Distress Tolerance Scale was used to measure distress tolerance (56). The Chinese vision of distress tolerance has good reliability and validity among Chinese adults. The scale consists of 13 items, such as “For me, it’s intolerable to feel pain or discomfort.” The responses of participators were rated on a 5-Likert point scale ranging from 1 (strongly agree) to 5 (strongly disagree). Higher scores indicated higher Distress tolerance (57). Cronbach’s alpha was 0.82 in this study.

### 2.3 Statistical analysis

The data were analyzed in SPSS version 25.0 and PROCESS V3. 4.1. First, we tested the common method biases; Then, we conducted the descriptive statistics and correlation analysis to examine the demographic characteristics and the association between various variables; Next, we tested the mediation effect of life history strategy by PROCESS macro model 4; Finally, we tested simultaneously all the study hypotheses by model 8. Data were calculated based on average score of items. Bootstrapped confidence interval (CI; 5,000 bootstrap samples) for the effect was obtained. If zero is not included in the confidence interval, effects are significant. Moreover, we transformed gender and left-behind experience into dummy variables and included them as control variables in subsequent analyses. gender: 1= male, 0= female; left-behind experience:1=have left-behind experience; 0= have no left-behind experience.

## 3 Results

### 3.1 Common method bias test

To assess and rule out common method bias, Harman’s single-factor test was employed. The analysis identified 14 factors with eigenvalues greater than 1, and the first factor accounted for 15.98% of the total variance. This proportion was lower than 40% recommended by Podsakoff (58), indicating that there was no the interpretation confusion induced by common method bias in the data analysis of this study.

### 3.2 Descriptive statistics and correlation analysis

Because the sample size difference between genders was too large, we used Welch’s t -test, a method more suitable for analyzing differences in unequal samples (59), to analyze gender differences in each variable. The result showed that compared with men, women had higher bedtime procrastination (t = -3.25, p< 0.01), perceived stress (t = -3.75, p< 0.001), and lower distress tolerance (t = 2.04, p< 0.05). At the same time, the independent sample T-test showed that the experience of being left behind significantly reduced the score of life history strategy (t = -2.74, p< 0.01). Further, gender and the experience of being left behind has been reported to be related with sleep problem (14, 60). Therefore, these two variables were selected as covariates in the subsequent analysis using PROCESS macro for SPSS (61).

**Figure 2:**
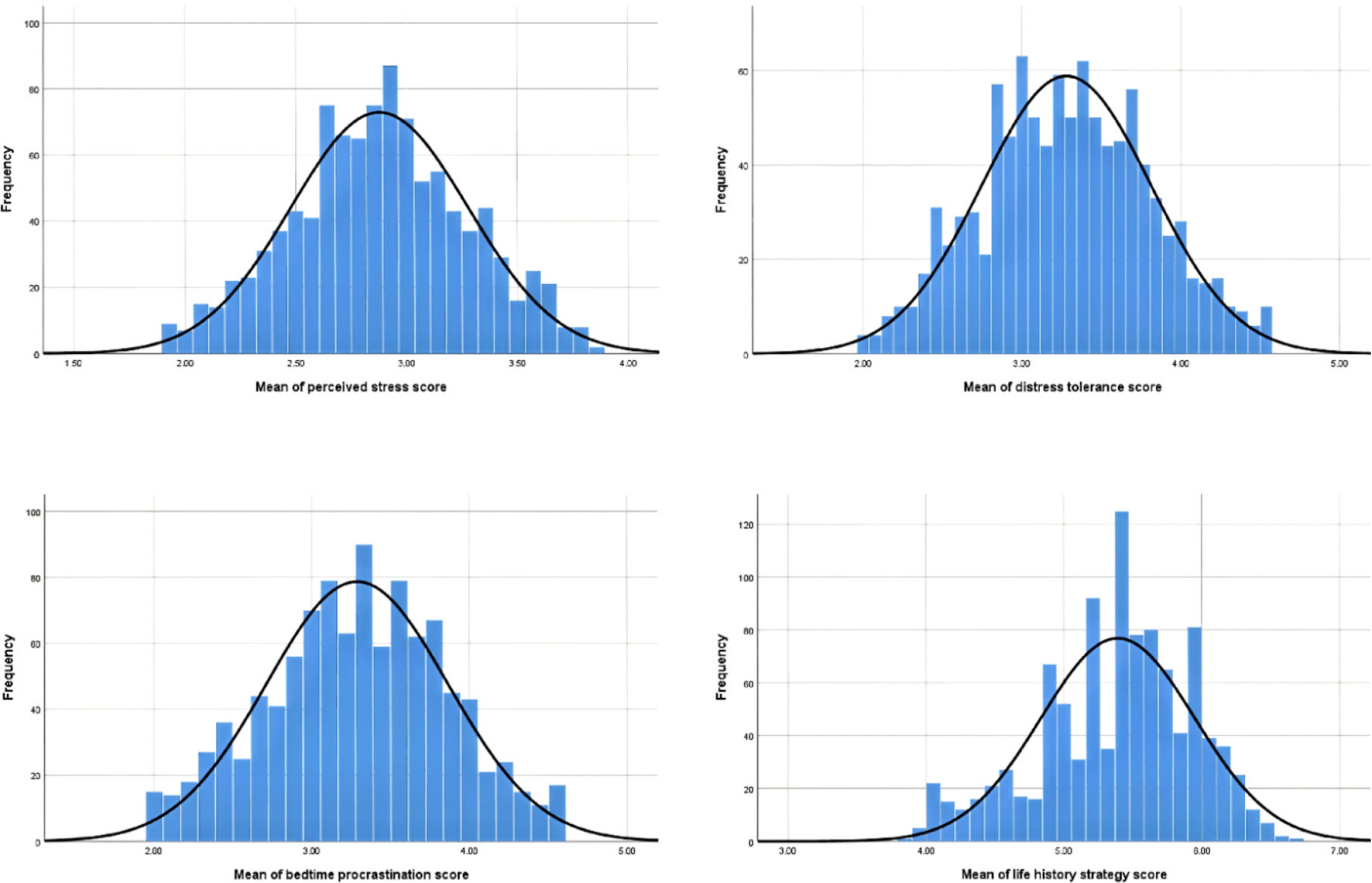
Tests for normal distribution of variables.

We tested the data for normal distribution and the results showed that it obeyed a normal distribution (see **Figure 2**). Then, to examine relationships among the main variables, Pearson’s correlation coefficients were calculated (see **Table 1**). Results showed that perceived stress was positively correlated with bedtime procrastination (r = 0.32, p < 0.001), negatively correlated with life history strategy (r = -0.40, p < 0.001) and distress tolerance (r = -0.46, p < 0.001). Distress tolerance was negatively correlated with bedtime procrastination (r = -0.25, p < 0.001), positively corrected with Life history strategy (r = 0.31, p < 0.001). Life history strategy was negatively correlated with bedtime procrastination (r = -0.33, p < 0.001).

**Table 1:** Descriptive statistics and correlations between variables.

Variable	*M ± SD*	1	2	3	4
1. Perceived Stress	2.88±0.40	1			
2. Distress Tolerance	3.28±0.53	-0.46^***^	1		
3. Life History Strategy	5.39±0.56	-0.40^***^	0.31^***^	1	
4. Bedtime Procrastination	3.29±0.58	0.32^***^	-0.25^***^	-0.33^***^	1

N=1021, ^***^ *p* < 0.001.

### 3.3 Testing for mediation effect

As Model 4 is suitable for simple mediation effect tests, it allows for comprehensive effect estimation and effective inclusion of control variables in the analysis, making the results more precise (62), we ran PROCESS macro-Model 4 to test hypothesis 1. Results showed that after controlling for covariates (gender and left-behind experience), life history strategy partly mediates the relationship between perceived stress and bedtime procrastination(R^2^ = 0.16, F = 46.79, p < 0.001). Specifically, the results showed that perceived stress negatively predicted on life history strategy (β = -0.57, p < 0.001. see Model 1 in **Table 2A**), while a lower LHS score indicates a tendency to use a faster life strategy. Thus, it means that the higher level of students’ perceived stress, the faster life history strategy was initiated. Perceived stress positively predicted on bedtime procrastination (β = 0.31, p <.001. see Model 2 in **Table 2A**), while life history strategy negatively predicted on bedtime procrastination (β = -0.25, p < 0.001.see Model 2 in **Table 2A**), suggesting that the faster life history strategy was initiated, the more bedtime procrastination. In summary, higher perceived stress induced faster life history strategy, which in turn led to higher bedtime procrastination. Moreover, the biased-corrected percentile bootstrap method was used to show that the indirect effect of perceived stress on bedtime procrastination through life history strategy was significant (ab=0.14, *SE*=0.02, and the 95%CI = [0.10, 0.19]), the direct effect of perceived stress on bedtime procrastination was significant (c’=0.31, *SE*=0.05, and the 95%CI = [0.22, 0.40]). Therefore, life history strategy partly mediates the relationship between perceived stress and bedtime procrastination. This means that perceived stress affect bedtime procrastination not only directly, but also indirectly through life history strategy. The percentage of this mediation effect of the total effect was 31.72%. These results support Hypothesis 1 (see **Tables 2A, B**).

**Table 2A:** Testing the mediation effect of Life History Strategy on Bedtime Procrastination.

Model 1 (Life History Strategy)					Model 2 (Bedtime procrastination)			
	*β*	*t*	*SE*	95%CI	*β*	*t*	*SE*	95%CI
Constant	7.11	59.82^***^	0.12	6.88~7.35	3.77	14.31^***^	0.26	3.25~4.29
Gender	-0.11	-3.15^**^	0. 03	-0.17~-0.04	-0.10	-2.89^**^	0.04	-0.17~-0.03
Left-behind experience	-0.09	-2.75^**^	0. 03	-0.15~-0.03	0.02	0.52	0.03	-0.05~0.08
PS	-0.57	-14.29^***^	0.04	-0.65~-0.49	0.31	6.68^***^	0.05	0.22~0.40
LHS					-0.25	-7.59^***^	0.03	-0.31~-0.18
*R^2^*	0.17				0.16			
*F*	71.85				46.79			

N = 1021. PS, perceived stress; LHS, life history strategy. ^**^ *p* < 0.01, ^***^ *p* < 0.001. β is the unstandardized coefficient.

**Table 2B:** The bootstrapping analysis of the mediating effects.

	Effect	*SE*	Boot CI lower	Boot CI upper
Total effect	0.45	0.04	0.36	0.53
Direct effect	0.31	0.05	0.22	0.40
Indirect effect	0.14	0.02	0.10	0.19

### 3.4 Testing for the moderated mediation model

**Figure 3:**
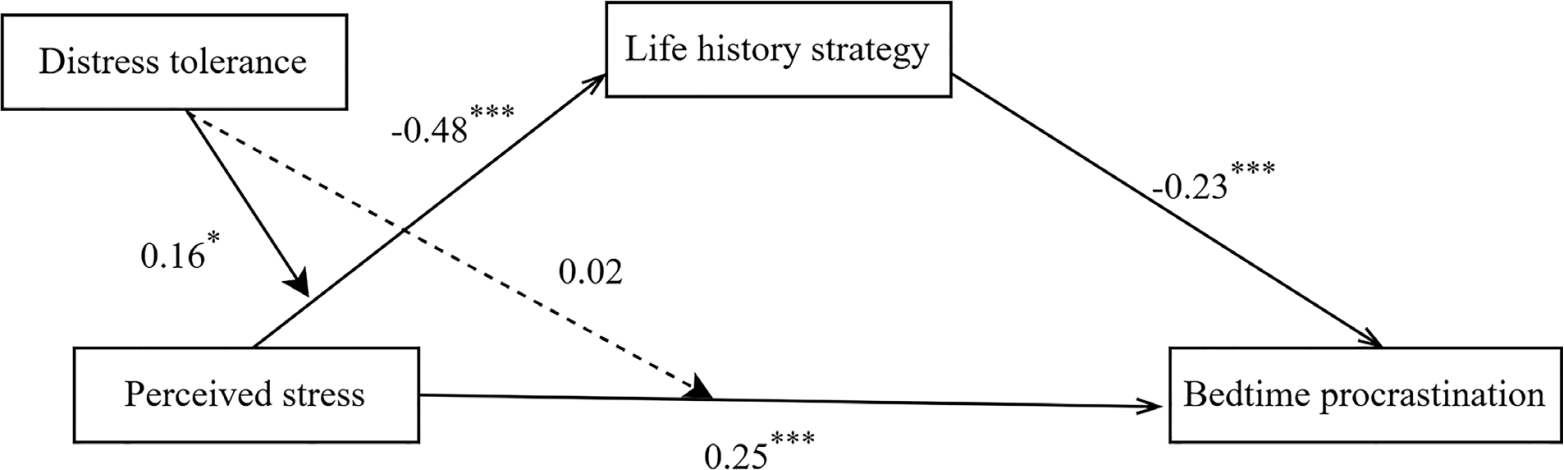
Path coefficients of the moderated mediation model. Notes of Figure In this model, We examined the significance of each path and labeled its coefficients, ^*^p< 0.05, ^***^p< 0.001. β is the unstandardized coefficient.

We ran the PROCESS macro model 8 to examine hypothesis 2 and hypothesis 3. Overall testing models are presented in **Figure 3**, and the specific indirect effects are presented in **Table 3A**. The results showed that perceived stress negatively predicted on life history strategy (β = -0.48, p < 0.001.see Model 3 in **Table 3A**), and the interaction effect of perceived stress and distress tolerance on life history strategy was significant (β = 0.16, p < 0.05.see Model 3 in **Table 3A**). Therefore, the effects of perceived stress on life history strategy are moderated by distress tolerance, and Hypothesis 2 is supported. Perceived stress positively predicted bedtime procrastination (β = 0.25, p < 0.001. see Model 4 in **Table 3A**), but the interaction effect of perceived stress and distress tolerance on bedtime procrastination was not significant (β = 0.02, p > 0.05.see Model 4 in **Table 3A**). That is to say, the direct effects of perceived stress on bedtime procrastination are not moderated by distress tolerance, the Hypothesis 3 was not supported. Therefore, our hypothetical model was partially supported (see **Figure 3**), and the index of moderated mediation was -0.038, *SE* = 0.018, 95% CI = [-0.073, -0.003] (see Model 5 in **Table 3A**).

**Table 3A:** Testing for the moderated mediation model.

Model 1 (Life History Strategy)					Model 2 (Bedtime Procrastination)			
	*β*	*t*	*SE*	95%CI	*β*	*t*	*SE*	95%CI
Constant	5.48	219.19^***^	0.03	5.43~5.53	4.58	25.02^***^	0.18	4.22~4.94
Gender	-0.11	-3.26^**^	0.03	-0.18~-0.04	-0.10	-2.82^**^	0.04	-0.17~-0.03
Left-behind experience	-0.09	-2.80^**^	0.03	-0.15~-0.03	0.02	0.56	0.03	-0.05~0.09
PS	-0.48	-10.7^***^	0.04	-0.57~-0.39	0.25	5.10^***^	0.05	0.16~0.35
DT	0.17	5.04^***^	0.03	0.10~0.23	-0.10	-2.73^**^	0.04	-0.17~-0.03
PS×DT	0.16	2.17^*^	0.07	0.02~0.31	0.02	0.21	0.08	-0.14~0.17
LHS					-0.23	-7.10^***^	0.03	-0.30~-0.17
*R2*	0.20				0.16			
*F*	50.01				32.64			
Model 3								
Index of moderated mediation	*β*	*SE*	*95%CI*					
	-0.038	0.018	-0.073~-0.003					

N=1021. PS, perceived stress; DT, distress tolerance; LHS, life history strategy. ^*^p < 0.05,^**^p < 0.01, ^***^p < .001. β is the unstandardized coefficient.

**Figure 4:**
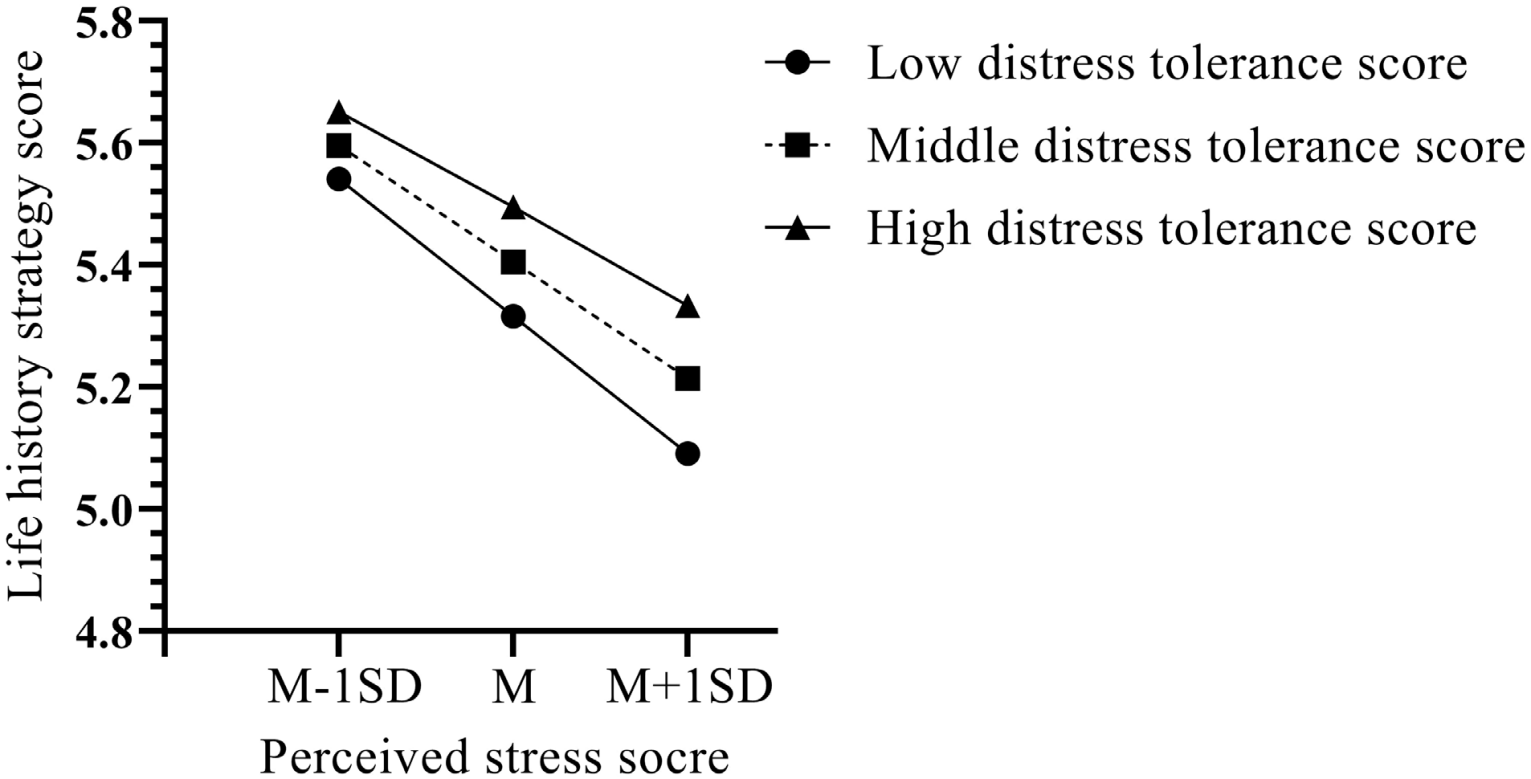
Interaction effect of distress tolerance and perceived stress on life history strategy. Notes of Figure High and low scores for perceived stress and distress tolerance are one standard deviation above and below the mean, respectively. A higher life strategy score indicates a tendency to use a slower life strategy; conversely, a lower score indicates a tendency to use a faster life strategy. A higher distress tolerance score indicates a higher distress tolerance; conversely, a lower distress tolerance score indicates a lower distress tolerance.

We further conducted a simple slope analysis to explore the pattern of the moderating effect. The results indicated that perceived stress was negatively correlated with life history strategy for both adolescents with higher distress tolerance (Bsimple = -0.39, p < 0.001) and those with lower distress tolerance (Bsimple = -0.56, p < 0.001). However, bias-corrected percentile bootstrap analysis revealed that the relationship between perceived stress and life history strategy was stronger under low distress tolerance (β = 0.13, *SE* = 0.03, 95% CI = [0.09, 0.19]) than at the high level of distress tolerance (β = 0.09, *SE* = 0.02, 95% CI = [0.06, 0.13], as shown in **Table 3B**. As shown in **Figure 4**, as an individual moves from low to high perceived stress, the lower distress tolerance is associated with lower life history strategy score than higher distress tolerance level. Given that lower life history strategy scores indicate faster life history strategy, our data suggest that subjects with lower distress tolerance tend to use faster life history strategy as the perceived stress increase.

**Table 3B:** Conditional indirect effect of distress tolerance when life history strategy between perceived stress and bedtime procrastination.

Mediator	Distress tolerance	Effect	*SE*	Boot CI lower	Boot CI upper
Life history strategy	M-SD	0.13	0.03	0.09	0.19
	M	0.11	0.02	0.07	0.15
	M+SD	0.09	0.02	0.06	0.13

## 4 Discussion

The role of stress in inducing bedtime procrastination is a new research topic that has emerged in recent years. However, the underlying psychological mechanism of this phenomenon is still unclear. Perceived stress refers to the imbalance between perceived environmental demands and their ability to cope with those demands (32). When an individual believes that his or her abilities are adequate to cope with environmental demands, perceived stress is low. Conversely, when an individual believes that his or her abilities are insufficient to cope with environmental demands, perceived stress is high. A previous study found that self-efficacy, a subjective experience of the ability to cope with stress, was negatively correlated with bedtime procrastination, and harm avoidance mediated the relationship between self-efficacy and bedtime procrastination (34). In fact, when environmental demands exceed an individual’s ability to cope, harm avoidance behavior has important survival adaptation significance. Therefore, bedtime procrastination may be a positive and proactive adaptive behavior under stressful situations, rather than a maladaptive behavior. From the perspective of life history strategy in evolutionary psychology, this is a fast life history strategy. The present study further explored this idea and found that life history strategies mediated the effect of perceived stress on bedtime procrastination. Furthermore, distress tolerance affected the indirect effect of perceived stress on bedtime procrastination, altering life history strategies by interacting with perceived stress.

### 4.1 The mediating role of life history strategies in the relationship between perceived stress and bedtime procrastination

The college students are in emerging adulthood. On the one hand, they are in the transition from school to society, with heavy study tasks and confusion about the future; on the other hand, their emotion regulation abilities are immature, despite struggling to hone their emotion regulation abilities. Thus, they often report high levels of perceived stress (63), and may be at risk for mental health problems (64). Consistently, university students had a higher incidence of bedtime procrastination than non-students in Polish (2); the present study found that perceived stress was significantly positively correlated with bedtime procrastination among college students in a Chinese sample. Further, our study extended this idea, finding that life history strategy mediates the relationship between these two variables.

As mentioned above, college students are in a period of heavy study tasks, confusion about the future, and immature emotion regulation skills. In this context, the future is usually full of uncertainty and difficult to predict. That is to say, it is uncertain whether investing in the future will pay off. For college students, it is crucial to resolve excessive psychological pressure from uncertainty about the future and low self-efficacy in stressful situations promptly. Therefore, they focus more on the present, less on the future, manifesting by immersing oneself in relaxing activities before bed, despite knowing there will be side effects on the longer time scale. Ultimately, these behaviors lead to bedtime procrastination. From the perspective of life history strategy, this fast life history strategy may help them to escape the aforementioned excessive emotional distress, is more adaptive. Many previous studies have shown the role of immersive activities before bed in bedtime procrastination (17, 65, 66). Our study provides a possible explanation for why they are prone to engage in these activities, which ultimately leads to bedtime procrastination.

### 4.2 The moderation of distress tolerance

Distress tolerance is one of the dispositions that underlie individual differences in emotion regulation abilities. Low distress tolerance is characterized by not accepting distress, perceiving it as unbearable and lower abilities to cope with distress than others. Individuals with low distress tolerance use the quickest and easiest methods to boost their mood, regardless of the side effects of their actions (56). Low distress tolerance works in favor of avoidance tendency that prevail in form of procrastination. Therefore, distress tolerance might regulate bedtime procrastination by interacting with perceived stress through two pathways: (1) the indirect effect of perceived stress on the bedtime procrastination through the life history strategy; (2) the direct effect of perceived stress on the bedtime procrastination. Our results support the former hypothesis, but not the latter. This is consistent with previous findings that distress tolerance modulates perceived stress and life history strategies in other forms of disorders (67). The results of the present study further illustrate the role of life history strategy in perceived stress leading to bedtime procrastination. Distress tolerance interacts with perceived stress to further exacerbate bedtime procrastination by altering life history strategy. Therefore, distress tolerance may be an effective potential intervention target for bedtime procrastination. On the other hand, to our knowledge, research on how distress tolerance affects bedtime procrastination is still very rare, their relationship still needs further in-depth exploration.

The present study has the following theoretical and practical contributions. First, it theoretically extends our understanding about the psychological mechanism underling perceived stress inducing bedtime procrastination in a life history framework. Bedtime procrastination might be an active adaptive behavior rather than a passive result of a failure of self-control, particularly in the stressful situations. Distress tolerance did not directly moderate the relationship between perceived stress and bedtime procrastination, but rather indirectly through life history strategies. Secondly, practically the present study suggested that life history-based interventions might be an effective intervention for stress-induced bedtime procrastination. Specifically, it might be helpful for alleviation of bedtime procrastination to develop slow life history strategy, when faced with similar stressful situations in the future. Further, the present study also suggested that distress tolerance skills training might be another potential effective method to relieve bedtime procrastination triggered by perceived stress indirectly by developing slow life history strategy.

### 4.3 Limitations of this study

Several limitations need to be considered when interpreting the findings. First, our cross-sectional data limited causal inferences. It is necessary to use longitudinal designs to obtain stronger empirical evidence of causal evidence in future research. Second, a questionnaire was used to measure the subjects’ bedtime procrastination, the self-reported method is somewhat subjective and can play an impact on the results of the study. Future studies should enrich the measurement methods to increase objective indicators, for example, by using a bracelet to record the subjects’ sleep circumstances. Finally, although the model hypothesized in this study is partially supported, the effect size is not large. This may be due to few control variables in this study. In addition, it suggests that there are other important factors besides perceived stress that have an impact on bedtime procrastination and future research should attempt to control for them.

## 5 Conclusion

In summary, we revealed the relationship between perceived stress and bedtime procrastination of college students in the framework of life history strategy and found that life history strategy serves as a mediating role between them. Further, distress tolerance moderated the indirect effect between them mediated by life history strategy, but not the direct effect between them.

## Data Availability

All data generated or analyzed during this study are included in the article/**Supplementary Material**. For further inquiries, please contact the corresponding author.
